# Development and psychometric testing of nursing students’ perceptions of clinical stressors scale: an instrument design study

**DOI:** 10.1186/s12888-020-02964-8

**Published:** 2021-01-02

**Authors:** Foozieh Rafati, Hamid Sharif Nia, Zohreh Khoshnood, Kelly-Ann Allen

**Affiliations:** 1 Department of Nursing, Jiroft University of Medical Sciences, Jiroft, Iran; 2 Department of Nursing, Mazandaran University of Medical Sciences, Sari, Iran; 3 Department of Community Health Nursing, Razi Faculty of Nursing and Midwifery, Kerman University of Medical Sciences, Kerman, Iran; 4 The Faculty of Education, Monash University, Clayton, Australia

**Keywords:** Nursing students, Workplace stress, Reliability and validity, Factor analysis

## Abstract

**Background:**

In clinical environments, nursing students experience a range of stressors that can affect their health, learning, and quality of patient care. This study aimed to develop a Nursing Students’ Perceptions of Clinical Stressors Scale (NSPCSS) and to evaluate its psychometric properties.

**Methods:**

This exploratory, sequential mixed-method study was conducted in 2 phases. In the qualitative (item generation) phase, NSPCSS items were generated using the data collected from semi-structured interviews and a literature review. In the quantitative (psychometric evaluation) phase, face, content, construct, convergent, and discriminant validity and reliability of the scale were tested. To evaluate construct validity, exploratory and confirmatory factor analyses were performed on the data collected from 430 nursing students. Reliability was also assessed through internal consistency and composite reliability.

**Results:**

In this study, 6 factors were extracted from 30 itemes through exploratory factor analysis: (1) instructor’s limited competence in clinical environments, (2) inappropriate clinical environment, (3) inadequate knowledge and skills, (4) inefficient education in clinical planning, (5) instructor’s inappropriate conduct, and (6) concerns about the characteristics of nursing career. These factors accounted for 58.8% of the total variance. The results of the confirmatory factor analysis suggested the goodness-of-fit indices was acceptable. Furthermore, the internal consistency and composite reliability indices of all factors were greater than 0.7.

**Conclusions:**

The NSPCSS is a valid and reliable instrument for assessing clinical stressors among nursing students.

**Supplementary Information:**

The online version contains supplementary material available at 10.1186/s12888-020-02964-8.

## Background

Clinical education, which is a central part of most tertiary level training in nursing programs [1], can have significant effects on student outcomes related to professional learning and competency. The aim of clinical education is to enhance students’ professional knowledge and skills and to provide them with the opportunity to translate their knowledge into practice [2]. Clinical education also enables nursing students to face practical realities that may influence future professional practice [3]. Positive experiences in the clinical setting can enhance nursing students’ critical thinking and problem-solving abilities, promote professional attachment and self-confidence, and help them develop professional identity and professionalism [4]. In contrast, negative clinical experiences can affect nursing students’ self-confidence, satisfaction with nursing, preparedness for practice, and retention [5]. Although clinical education is an essential component of nursing education, students often perceive it as extremely stressful [6].

Despite different definitions of stress, there is lack of consensus about how it is best conceptualised [7]. For instance, stress has been defined as any biological response to an extrinsic or intrinsic stimulus [8]. However, psychological stress cannot be described in terms of stimulus-response alone [9]. Rather, individuals may experience stress when they perceive an event or situation is beyond their coping resources [10]. Hence, stress perceived by nursing students in a clinical environment is defined as the gap between students’ needs in a specific clinical situation and their resources or ability to cope with a task or situation [11].

Nursing students experience higher stress levels compared to students in other healthcare-related fields [12]. Research also shows almost all nursing students will experience moderate to high levels of stress when working in a clinical environment [13]. Also, the prevalence of stress among nursing students has been on the rise [14]. Major stressors reported by nursing students include limited knowledge and skills [11], fear of causing harm to patients [15], heavy workload [16], instructors’ incivility [14], observation by teachers and staff [17], and ineffective organization of clinical courses [18]. Clinical stress can impair students’ clinical performance, affect the quality of nursing care, endanger physical and mental health, and lead to job burnout [6]. Therefore, clinical stressors need to be effectively managed [19].

Effective stress management requires the accurate identification of stress [20]. Also, this identification is crucial for planning future health resources as well as teaching and learning [1]. Different instruments are used to assess the level of stress experienced by nursing students, and the most common of which is the Perceived Stress Scale [21]. It contains 29 items that are grouped into 6 areas: stress related to patient care, instructors and staff, assignments and workload, peers and daily life, lack of professional knowledge and skills, and environmental factors [21]. This scale is developed to assess perceived stress and severity during the first clinical experience of nursing students, and thus not applicable for all nursing students. Another instrument used to assess stress experienced by nursing students is the Student Nurse Stress Index, which contains 22 items with 4 subscales: academic load, clinical concern, interface worries, and personal problems [22]. The Stress in Nursing Students Scale, which contains 43 items, is also used to measure the level of stress among nursing students. The scale presents a different structure and conceptualization of subscales (e.g., clinical stressors, confidence, education, and finance) [23]. Yoo et al. developed a stress scale for Korean nursing students with 58 items through literature review [24]. However, the latter 3 tools are not specifically designed to assess stress related to clinical settings. A longer 60-item instrument, with 4 subscales including interpersonal relationships, humiliating experiences, educational environment clinical experiences, and unpleasant feelings, has also been used to assess stress among nursing students [25]. This instrument was not evaluated psychometrically or based on empirical evidence. Moreover, the large number of questions may be burdensome to complete. Gibbons et al. developed a questionnaire to asses distress and eustress in nursing students [26], however the questionnaire was developed specifically to assess stress among older adult nursing students [1], not among undergraduate students.

Existing questionnaires in the field do not have the necessary comprehensiveness to measure stress in undergraduate nursing students or measure the general clinical stressors of undergraduate nursing students more generally. Therefore, due to the importance of the subject, this study was conducted with the aim of developing and psychometrically testing the Nursing Students’ Perceptions of Clinical Stressors Scale (NSPCSS).

## Methods

### The aim

This study was conducted with the aim of developing and psychometric testing of the Nursing Students’ Perceptions of Clinical Stressors Scale.

### Design

This exploratory, sequential mixed-method study was conducted in both qualitative and quantitative phases.

### Qualitative phase

In the qualitative phase, the NSPCSS (Nursing Students’ Perceptions of Clinical Stressors Scale) items were generated using the data from interviews with 19 nursing students and a literature review. Accordingly, in-depth semi-structured interviews were held for freshman to senior nursing students who were selected purposively from 4 nursing schools in southeast of Iran.

The students who successfully completed at least 1 clinical course were included in the study. The participants were recruited with maximum demographic variation in terms of age, gender, academic year, and grade point average. The interviews were held at the participants’ school or dormitory. During interviews, the students were asked to describe their experience of clinical stressors. Each interview started with a open and broad question (Please describe your experience of stressors in clinical setting.). Exploratory questions were asked whenever necessary (Can you explain more? Can you give an example?). Each interview was ended with the following question: Was there a question that I should have asked but did not? The length of each interview was 60–90 min.

**Fig. 1:**
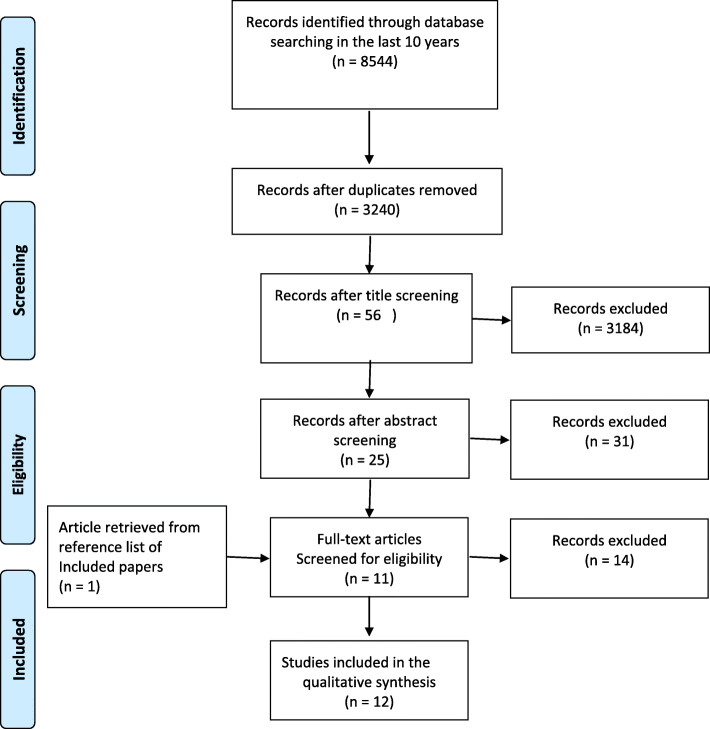
PRISMA flowchart

To review the literature, Google Scholar, PubMed, ScienceDirect, Scopus, SID, and Magiran were searched using the following keywords: “clinical stress”, “clinical stressors”, “nursing student”, “stress-inducing factors”, “stressor”, “clinical training”, “clinical environment”, and “clinical setting.” The review process was done on peer-reviewed articles published in the last 10 years. The retrieved articles were assessed for complementing the data collected in the qualitative phase (Fig. 1).

Using the data from the interviews and the literature review, an item pool was generated. None of the items had negative wording. The items were scored on a 5-point Likert scale of 1 (not important at all) to 5 (completely important).

### Quantitative phase

In the quantitative phase, the face, content, construct, convergent, and discriminant validity, internal consistency, and composite reliability (CR) of the NSPCSS were evaluated.

### Face validity evaluation

Qualitative and quantitative methods were used for the face validity evaluation. In the qualitative evaluation of face validity, face-to-face interviews were conducted with 12 nursing students. They discussed the comprehensiveness, appropriateness, clarity, and necessity of each item. The items were then revised based on the comments.

In the quantitative evaluation of the face validity, 12 students at different educational levels were asked to rate the importance of each item on a 5-point scale from 1 (not important) to 5 (very important). The impact score of each item was calculated by multiplying its importance score by the number of students who had rated it 4 or 5, and items with impact scores of lower than 1.5 were removed [27].

### Content validity

Content validity was assessed using both qualitative and quantitative methods. For a qualitative content-validity evaluation, 12 nursing instructors who were experienced in instrument development were asked to comment on the grammar, wording, item allocation, and scoring of the NSPCSS items. The scale was revised based on their comments.

Next, the validity of the instrument was evaluated using content validity ratio (CVR) and content validity index (CVI). Accordingly, 15 nursing instructors were asked to rate the necessity of each NSPCSS item on a 3-point scale: “necessary” (score of 1), “useful but not necessary” (score of 2), or “not necessary” (score of 3). Then, the answers were calculated based on the following formula: $CVR=\frac{nE-N/2}{N/2}$ (nE = the experts who selected the necessory option, *N* = total number of experts). The items with CVR values lower than 0.49 were removed according to Lawshe table.

For the CVI calculation, the same experts were asked to rate the relevancy of each item to nursing students’ stressors in a clinical setting on a 4-score spectrum (1. not relevant; 2. somewhat relevant; 3. quite relevant; and 4. highly relevant). We determined the ICVI by dividing the number of experts who had rated the item as “3” or “4” by the total number of experts. Value items CVI (I-CVI) with values greater than 0.7 were considered acceptable. Moreover, an average scale-level CVI (S-CVI/Ave) was evaluated by averaging the I-CVI scores. An S-CVI/Ave of greater than 0.9 was considered acceptable [27].

### Primary reliability evaluation

Before construct validity testing, 30 students were asked to complete the NSPCSS. Their responses were used to assess the internal consistency of the scale. The items with an interitem correlation value of less than 0.3 were removed.

### Construct validity evaluation

As a rule of thumb, a sample of 200 persons is adequate for a construct validity evaluation [28]. Therefore, 430 nursing students were selected to complete the NSPCSS for exploratory and confirmatory factor analyses. The questionnaire contained demographic information (eg, age, gender, educational semester, and clinical experience) and 37 initial NSPCSS items.

The sampling adequacy for the exploratory factor analysis (EFA) was assessed using the Kaiser-Meyer-Olkin and Bartlett tests. Then, the latent factors of the NSPCSS were extracted via the maximum-likelihood EFA with a Promax Rotation. Parallel analysis was used to determine the number of extractable factors. Also, the minimum acceptable factor loading value for the presence of an item in a factor was 0.25, which was calculated using this equation:

$$ CV=5.125\div \sqrt{\left(\mathrm{n}-2\right)}. $$

Based on the 3-indicator rule, each factor had to have at least 3 items [29]. The items with communality values lower than 0.2 were removed [30]. With a confirmatory factor analysis (CFA), the extracted factor model was evaluated via maximum likelihood estimation using the following model fit indices: root means score error of approximation (RMSEA), comparative fit index (CFI), parsimony comparative fit index (PCFI), the goodness-of-fit index (GFI), adjusted goodness-of-fit index (AGFI), minimum discrepancy function divided by degrees of freedom (CMIN/DF), normed fit index (NFI), and parsimony normed fit index (PNFI).

### Normal distribution, outliers, and missing data

Univariate normality was checked using skewness (±3) and kurtosis (±8). Multivariate outliers were assessed using the Mahalanobis d-squared test (*P* < 0.001). In addition, the multivariate normality was evaluated using the Mardia’s coefficient of multivariate kurtosis (< 20) [31]. Missing data were assessed via multiple imputations and were replaced via the mean of participants’ scores.

### Convergent and discriminant validity

Convergent and discriminant validity of the instrument were evaluated through Fornell and Larcker’s approach using the average variance extracted (AVE), maximum shared squared variance (MSV), and CR. The convergent validity is confirmed if the items of the intended scale show strong correlations.

In addition, discriminant validity is supported when the extracted factors are distinct from each other [32]. To confirm convergent validity, AVE should be greater than 0.5 and the CR should be greater than the AVE. However, discriminant validity is maintained if AVE is greater than the MSV [33].

### Reliability

To assess the internal consistency of the instrument, Cronbach’s alpha, McDonald’s omega, and Average Inter-Item Correlation (AIC) were calculated [34]. An acceptable internal consistency is ensured with a coefficient greater than 0.7 and an AIC between 0.2 and 0.4 [29]. The CR is the substitute for Cronbach’s alpha in structural equation modeling [35].

### Scoring

The NSPCSS items were scored on a linear 5-point Likert scale.

### Setting and sample

The study was conducted on undergraduate nursing students in the southeast of Iran. For sampling in the qualitative phase, we used a purposeful sampling method to select participants among undergraduate nursing students (the second semester to the eighth semester). Sampling continued until data saturation, which means no new code was extracted [36]. In this survey, we achieved data saturation after interviewing 17 participants; however, we selected 2 additional participants to guarantee data saturation. Thus, 19 nursing students participated in the qualitative phase. The quantitative phase was conducted on 430 nursing students using census sampling method from 4 nursing schools in southeast of Iran. The inclusion criteria included being a nursing student, passing at least 1 clinical course, and having no history of physical or psychological disease.

### Ethical considerations

The Ethics Committee in Biomedical Research of Jiroft University of Medical Sciences approved this study (code: IR.JMU.REC.1397.030). Before conducting the study, its objectives were explained to the participants and they were assured of the confidentiality of their data. Informed consent was also obtained for interviews.

### Data analysis

The qualitative data were analyzed using the conventional content analysis approach proposed by Graneheim and Lundman [37], in which each interview was transcribed immediately and read several times until a general impression was received. Then, all texts were read line-by-line and were broken down into meaning units, which were key phrases in the text. The condensed meaning units were condensed and labeled with codes. Next, the codes were allocated into subcategories based on similarities and differences. Similar subcategories were grouped into main categories. Finally, themes were determined as the expression of the latent meaning of the text. Data were managed by MAXQDA 12 software. Also, trustworthiness was ensured via 4 criteria: credibility, dependability, confirmability, and transferability [36].

F.R and Z.Kh. separately assessed the papers that were included based on the literature review. Then, each of them extracted codes related to the clinical perceived stress of undergraduate nursing students and imported them into a table. Then, they reviewed the codes for duplication, overlap, and relativeness. Finally, the codes extracted from reviewing the texts were merged with those extracted from the interviews. The quantitative data were analyzed using the SPSS-AMOS24 and the SPSS R-menu2.0.

## Results

### Item generation

We extracted 151 codes in 11 main categories by analyzing the data gathered by interviews. The identified categories were related to students’ perceived stress in clinical environment, patients’ care, instructors and nursing staff, assignments and workload, peers and daily life, a lack of professional knowledge and skills, environmental factors, interpersonal and relational factors, the characteristics of nursing, nursing curriculum, limited perceived support, and shameful experiences.

Also, 3 categories were identified through the review of the literature: academic stressors (eg, assignments, workload, exams, fear over failure, and relationships with university staff), clinical stressors (eg, death of a patient, lack of professional knowledge and skills, emergency clinical situations, and relationships with clinical staff), and external stressors (eg, financial stressors and daily life problems). Our literature review indicated clinical stressors were more dominant than academic and external stressors. Thirty-four extracted codes related to clinical stressors category were added to the item pool.

Therefore, during the item generation phase, we extracted 185 codes in 11 categories through qualitative content analysis on 19 interviews and litearature review. A further refinement of the items reduced them to 61 items that were grouped into the following categories: stress related to patient care, stress related to instructors and nursing staff, stress related to assignments and workload, stress related to peers and daily life, stress related to a lack of professional knowledge and skills, stress related to environmental factors, stress related to interpersonal and relational factors, stress related to the characteristics of nursing, stress related to nursing curriculum, stress related to limited perceived support, and stress related to shameful experiences.

### Face and content validity

Five items were revised in qualitative face-validity evaluations. Moreover, 2 items (items 36 and 34) with an impact score of smaller than 1.5 (Table 1) were revised in the quantitative face validity evaluation. After face validity evaluations, the previously mentioned 61 items were grouped into stress related to the following 6 categories: patient care (6 items), instructors and nursing staff (26 items), heavy workload and intraprofessional relationships (7 items), environmental factors (7 items), interpersonal and relational factors (9 items), and the characteristics of nursing and nursing curriculum (6 items).

**Table 1:** Factors extracted from NSPCSS

Factors	Items	Factor loading	*h2*	*λ*	Variance
Instructors Limited Clinical Competence	9. Instructor’s inadequate attention and guidance	.926	.775	6.463	%17.94
	8. Difference between instructor’s education and student’s educational needs	.902	.798		
	15. Instructor’s limited skills	.873	.727		
	10.Instructor’s use of traditional teaching methods and routine in clinical education	.858	.728		
	2.Instructor failure to provide independence for students	.832	.680		
	13. Over emphasis of theoretical training (as opposed to applied clinical education by instructor)	.776	.648		
Inappropriate Clinical Environment	24. Inadequate equipment for appropriate nursing care	.740	.440	4.961	%14.25
	22. Shortage of recreational and educational facilities in the clinical environment	.723	.410		
	23.Observing the violation of patient rights by healthcare providers	.686	.453		
	6. Students exploitation by healthcare providers	.542	.359		
	7.Observing non-standard care delivery to a patient by others	.536	.404		
	21. Inadequate time for appropriate nursing care	.490	.310		
	20. Fatigue due to heavy physical workload	.445	.357		
	28. Receiving inadequate support from healthcare providers	.442	.406		
	25. Misconduct by a patient or family member	.334	.231		
	4. Inconsistency between the theoretical and clinical education explanation provided.	.307	.266		
Inadequate Knowledge and Skills	2.Student’s inadequate knowledge for patient care	.949	.915	3.249	%9.03
	1.Student’s inadequate experience in patient care	.843	.754		
	3.Student’s inadequate skills for patient care and equipment use	.799	.615		
Inefficient Clinical Education Planning	36. Vague job description	.884	.714	2.864	%6.82
	35. Vague explanations of the objectives of clinical education	.754	.626		
	37. Instructors’ personalized approach to the use of educational rules and regulations	.539	.502		
	34.Inappropriate planning for clinical education by school authorities	.473	.384		
Instructor’s Inappropriate Conduct	16.Instructor’s inappropriate conduct in the case of student error	.709	.452	2.732	%6.38
	17.Instructor’s high expectations	.652	.440		
	19.Instructor’s unfair evaluation	.587	.446		
	14.Lack of instructor’s feedback after doing a task	.447	.396		
	11.Instructor’s insufficient education about personal safety	.398	.341		
	18.Feeling of bafflement due to contradiction by some instructors	.305	.409		
Concerns over the Characteristics of Nursing	30.Concern over affliction of psychological problems during patient care	.793	.632	1.419	%4.43
	31.Concern over legal problems due to negligence or error in patient care	.649	.490		
	32.Concern over affliction of physical problems during patient care	.608	.491		

***Abbreviation*:** *ʎ* Eigenvalue, *h*^*2*^ communality

Seven items with CVR values less than 0.49 were removed via content validity evaluation by 15 experts. Moreover, items with CVI values less than 0.79 were revised. Therefore, the S-CVA/Ave of the 54-item NSPCSS was 0.97.

### Primary reliability evaluation

Thirty students completed the 54-item NSPCSS and the Cronbach’s alpha value was estimated to be 0.91. In addition, 17 items with the interitem correlation coefficients smaller than 0.3 were excluded and 37 items remained on the scale for further psychometric evaluation.

### Construct validity

In total, 430 students completed the NSPCSS for factor analysis. The students’ mean age was 21.58 ± 2.35, and most of them were female (229 students, 53.4%) and single (364 students, 84.7%). The response rate was 100%.

An EFA was performed on the data obtained from 215 students. The Keiser-Meyer-Olkin test value was .92 and Bartlett’s test value 6674.18 (*P* < .001). Six main factors were extracted using a parallel analysis: the instructor’s limited competence in clinical environments (6 items), inappropriate clinical environment (10 items), inadequate knowledge and skills (3 items), inefficient clinical education planning (4 items), inappropriate conduct by the instructor (6 items), and concerns about the characteristics of nursing (3 items), (Five items were removed based on factor loading in EFA). The eigenvalues of these 6 factors were 6.46, 4.96, 3.24, 2.86, 2.72, and 1.41, respectively, and they explained 58.8% of the total variance of the NSPCSS (Table 1).

**Fig. 2:**
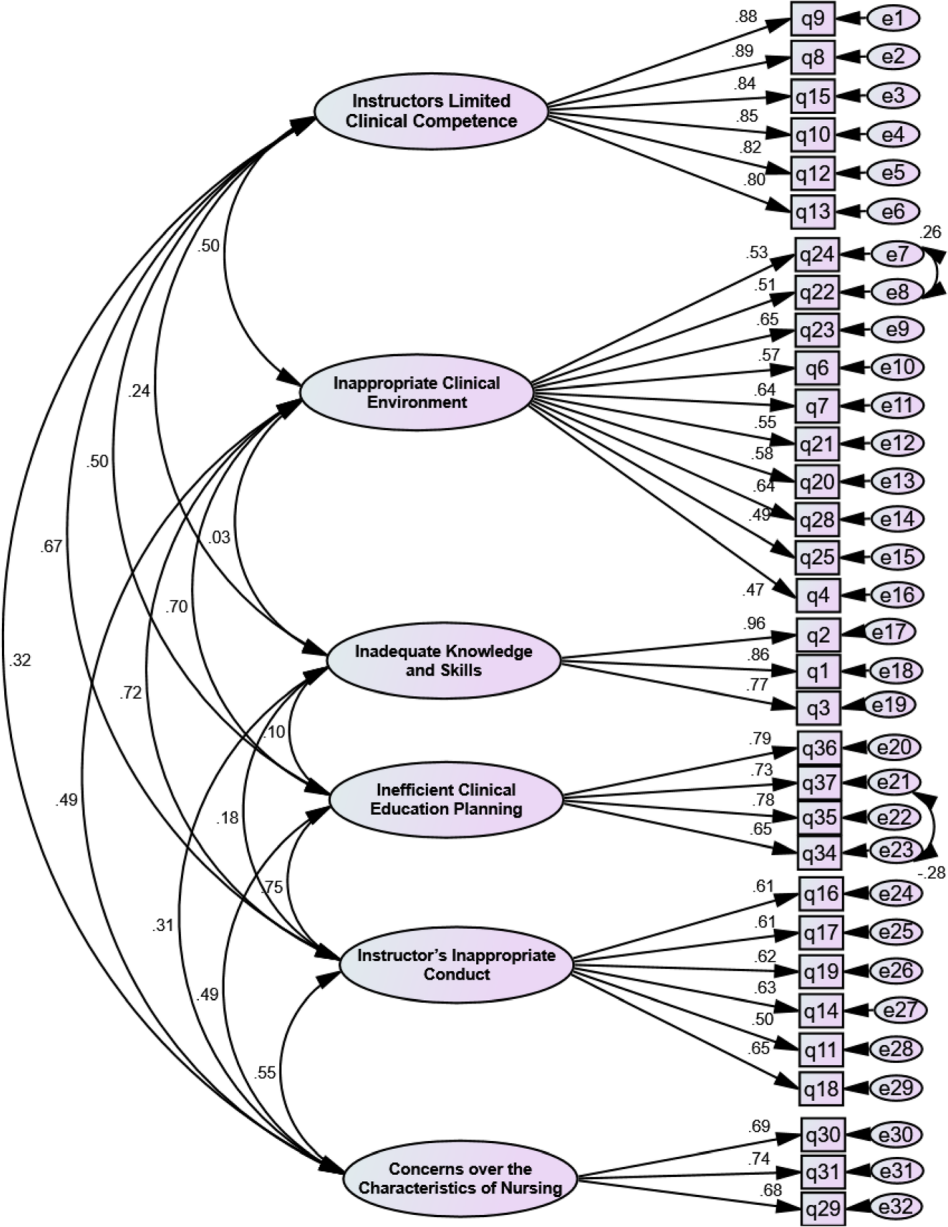
The confirmatory factor analysis model of NSPCSS

The extracted factor structure was evaluated using CFA and the data obtained from 215 students. Two items (4 and 25) were deleted in this phase. After a model correction that determined the correlation between measurement errors (between items 22 and 24 and items 34 and 37), the Chi-square index for the goodness-of-fit was calculated to be 742.87 (*n* = 210; *P* < 0.001). The other goodness-of-fit indices calculated were as follow: PCFI = 0.84, PNFI = 0.79, CMIN/DF = 1.91, RMSEA = 0.04, AGFI = 0.87, and IFI = 0.94. These indices confirmed the model’s goodness-of-fit (Fig. 2). All factor-loading values were greater than 0.5.

A CFA showed all items had a significant correlation with the factors. Moreover, the CR in all factors was greater than the AVE. The AVE of factors 1, 3, and 6 was greater than the MSV, and the discriminant validity of the NSPCSS was confirmed. Internal consistency and CR indices of all factors were greater than 0.7, confirming the acceptable internal consistency and reliability of the factors (Table 2).

**Table 2:** Convergent validity, discriminant validity, and reliability indices of NSPCSS

Index Factor	AVE	MSV	MaxR(H)	CR	Alpha	AIC	Omega
Instructors Limited Clinical Competence	.719	.453	.942	.931	.938	.718	.939
Inappropriate Clinical Environment	.347	.483	.812	.809	.824	.319	.826
Inadequate Knowledge and Skills	.752	.094	.944	.900	.894	.743	.900
Inefficient Clinical Education Planning	.552	.561	.839	.830	.814	.523	.820
Instructor’s Inappropriate Conduct	.366	.561	.779	.775	.774	.364	.776
Concerns over the Characteristics of Nursing	.748	.301	.750	.748	.746	.496	.748

***AVE*** Average Variance Extracted, *MSV* Maximum Shared Squared Variance, *CR* Composite reliability, *Alpha* Cronbach’s alpha, *AIC* Average Inter-item Correlation, *Omega* McDonald’s omega coefficient

## Scoring

The 30 items of the NSPCSS were scored on a 5-point scale. Therefore, the total score of the scale can range from 30 to 150 (30–59 = low, 60–89 = moderate, 90–119 = high, 120–150 = very high).

## Discussion

This study aimed to develop a NSPCSS and evaluate its psychometric properties. The findings of the study suggested that the scale is valid and reliable. The NSPCSS contains 30 items grouped in 6 subscales: The instructor’s limited clinical competence, inappropriate clinical environment, inadequate knowledge and skills, inefficient clinical education planning, inappropriate conduct by the instructor, and concerns about the characteristics of nursing. The acceptable explained variance of the scale confirms its ability to measure the concept of perceived stress among nursing students in clinical environments.

The first subscale extracted in the exploratory factor analysis was the instructor’s limited competence in clinical environments. This subscale accounted for the highest level of variance compared to other subscales. In line with this finding, inefficient educators have been identified as a primary concern in another qualitative study addressing nursing students’ clinical experiences [38]. It has also been demonstrated that encountering an inexperienced and incompetent instructor is perceived by nursing students as an unpleasant experience [39]. Students expect their instructors to organize their clinical courses, facilitate their learning, reduce the theory/research-practice gap, train them in evidence-based care, and empower them for clinical practice [40]. Since instructors are considered the most critical source of learning for students in a clinical environment [41], their limited knowledge and skills can be perceived by students as significant stressors. Thus, some strategies are required to both enhance instructors’ professional knowledge and skills and assess their performances. The items of “quality of tutorials” and “feeling stressed about the difference between instructors’ training and my expectations” were present in Gibbones^’^s et al. [20] and Sheu^’^s et al. [21] scale, respectively, which is in line with the findings of this study.

The second subscale of the NSPCSS was “inappropriate clinical environment”, the key components of which can be nurses’ negative attitudes towards nursing students, their limited support for other students, nurses and patients, diminished trust in students, and lack or inaccessibility of equipment (resulting in wasting time and delaying nursing care) [38]. “Stress from the environment” was a subscale in Shue^’^s et al. scale [21], although its items were different from the items in tools used in this study.

The third subscale of the NSPCSS was “inadequate knowledge and skills”. In a similar vein, other studies have shown that the lack of clinical knowledge and skills was the most common source of stress for nursing students [42]. The reason is that these students are afraid that their limited knowledge and skills could inflict injuries on patients. This subscale was developed in Shue et al. [21] study. Fear of failure [22], fear of making mistakes in the clinical environment [23], and lack of skills in providing care to the patient [25] were the stressors items for nursing students in other scales and were similar to findins of this study.

“Inefficient clinical education planning” was another subscale of the NSPCSS. Some of the main environmental stressors for nursing students in the clinical environment included an imbalance between the time of theoretical and clinical training courses, the incongruence between learning objectives, and the duration of clinical education [38], and vagueness of learning objectives and job descriptions. Nursing students in a previous study also reported that the nursing curriculum does not often consider their learning needs [43]. Some items of this subscale, eg, vagueness of learning objectives [23] and theory practice gap, are available in other nursing students’ stress assessment tools [21].

The fifth subscale of the NSPCSS was “inappropriate conduct by the instructor”. Behaviors such as inappropriate feedback, high expectations, and unfair student evaluations are stressful for nursing students. In an integrative review, Bhurtun et al. (2019) found that instructors were the strongest stressors for nursing students in clinical environments because the students felt the instructors were continuously observing and evaluating them [17]. There are also items related to the stress caused by clinical instructors in other tools [21, 24, 25].

The last subscale of the NSPCSS was “concerns about the characteristics of nursing”. Previous research has suggested that nursing students are mainly concerned about the transmission of communicable diseases [25]. They may also be concerned about occupational diseases due to the high prevalence of needle-stick injuries among nursing students [44]. Other studies have shown the high prevalence of health problems, including back pain [45] and mental health problems among nursing students [46].

Working in the nursing profession is associated with dangers that are always among the concerns of nursing students and are also seen in Moridi et al. [25] and Sheu et al. [21] studies.

The fit assessment of the extracted model (developed based on the literature review) demonstrated an acceptable fit with clinical stressors. In addition, the assessment of convergent and discriminant validity showed that all NSPCSS subscales had acceptable validity (convergent and discriminant). Convergent validity is confirmed if the items of the intended construct are close and share a large variance. However, this validity is not confirmed when the latent factors are inadequately explained by the extracted factors, and the items do not show strong correlations with each other [32]. Furthermore, discriminant validity is established when the items or the extracted factors of the construct in question are completely distinct from each other [47]. Taken together, the reliability evaluation of the NSPCSS showed that it is an empirically acceptable scale to use to evaluate the stress of nursing students in clinical settings.

The NSPCSS is a tool used to assess the level of stress experienced by nursing students. The strengths of this instrument include its development based on empirical data and existing literature and its construct validity assessment via both exploratory and confirmatory factor analyses. One of the limitations of the study involves nursing students’ close relationships with each other in clinical environments, which might have resulted in similar responses to NSPCSS items. Therefore, a psychometric evaluation of this instrument is recommended in different cultural and clinical contexts. Future research can also evaluate the tool in different clinical contexts in which student nurses work given the cross-cultural/international applicability for such an instrument.

## Conclusions

The NSPCSS is a comprehensive instrument with demonstrated validity and reliability that can be used to assess clinical stressors among nursing students. The tools designed in this study were used to assess the stress of nursing students in the clinical environment and design interventions and strategies to reduce stress, which will ultimately improve their clinical experience. Since the scale was developed based on the Iranian context, more studies are needed to support the adaptation of the NSPCSS in other contexts.

## Supplementary Information

**Additional file 1.**

## Data Availability

The datasets used and/or analyzed during the current study are available from the corresponding authors on reasonable request.

## Abbreviations

NSPCSS: Nursing Students’ Perceptions of Clinical Stressors Scale
PRISMA: Preferred Reporting Items for Systematic Reviews and Meta-Analyses
