# Supplementary material for: Development and psychometric testing of nursing students’ perceptions of clinical stressors scale: an instrument design study
Source: BMC Psychiatry. 2021 Jan 2;21:1. doi: 10.1186/s12888-020-02964-8 (PMC7777413; doi:10.1186/s12888-020-02964-8)
Supplement: Supplementary file 1 — Additional file 1. [file 12888_2020_2964_MOESM1_ESM.docx]

| Factors | Items | Always | often | sometimes | rarely | at all |
| --- | --- | --- | --- | --- | --- | --- |
| Instructors limited clinical competence | Instructor’s inadequate attention and guidance |  |  |  |  |  |
|  | Difference between instructor’s education and student’s educational needs |  |  |  |  |  |
|  | Instructor’s limited skills |  |  |  |  |  |
|  | Instructor’s use of traditional teaching methods and routine in clinical education |  |  |  |  |  |
|  | Instructor failure to provide independence for students |  |  |  |  |  |
|  | Over emphasis of theoretical training (as opposed to applied clinical education by instructor) |  |  |  |  |  |
| Inappropriate clinical environment | Inadequate equipment for appropriate nursing care |  |  |  |  |  |
|  | Shortage of recreational and educational facilities in the clinical environment |  |  |  |  |  |
|  | Observing the violation of patient rights by healthcare providers |  |  |  |  |  |
|  | Students exploitation by healthcare providers |  |  |  |  |  |
|  | Observing non-standard care delivery to a patient by others |  |  |  |  |  |
|  | Inadequate time for appropriate nursing care |  |  |  |  |  |
|  | Fatigue due to heavy physical workload |  |  |  |  |  |
|  | Receiving inadequate support from healthcare providers |  |  |  |  |  |
|  | Misconduct by a patient or family member |  |  |  |  |  |
|  | Inconsistency between the theoretical and clinical education explanation provided. |  |  |  |  |  |
| Inadequate knowledge and skills | Student’s inadequate knowledge for patient care |  |  |  |  |  |
|  | Student’s inadequate experience in patient care |  |  |  |  |  |
|  | Student’s inadequate skills for patient care and equipment use |  |  |  |  |  |
| Inefficient clinical education planning | Vague job description |  |  |  |  |  |
|  | Vague explanations of the objectives of clinical education |  |  |  |  |  |
|  | Instructors’ personalized approach to the use of educational rules and regulations |  |  |  |  |  |
|  | Inappropriate planning for clinical education by school authorities |  |  |  |  |  |
| Instructor’s inappropriate conduct | Instructor’s inappropriate conduct in the case of student error |  |  |  |  |  |
|  | Instructor’s high expectations |  |  |  |  |  |
|  | Instructor’s unfair evaluation |  |  |  |  |  |
|  | Lack of instructor’s feedback after doing a task |  |  |  |  |  |
|  | Instructor’s insufficient education about personal safety |  |  |  |  |  |
|  | Feeling of bafflement due to contradiction by some instructors |  |  |  |  |  |
| Concerns over the characteristics of nursing | Concern over affliction of psychological problems during patient care |  |  |  |  |  |
|  | Concern over legal problems due to negligence or error in patient care |  |  |  |  |  |
|  | Concern over affliction of physical problems during patient care |  |  |  |  |  |
